# A novel method for identifying coded tags recorded on aquatic acoustic monitoring systems

**DOI:** 10.1007/s10661-022-10500-2

**Published:** 2022-09-20

**Authors:** Christopher D. Lowe, Nicolas J. C. Tregenza, Claudia J. Allen, Georgina E. Blow, Hanna Nuuttila, Chiara M. Bertelli, Anouska F. Mendzil, Thomas Stamp, Emma V. Sheehan, Peter Davies, Jonathan C. D. Gordon, Jonathan D. Bolland, J. Robert Britton, Robert Main, Randolph Velterop, Charles Crundwell, Andrew Schofield, David R. K. Clarke

**Affiliations:** 1 Department of Bioscience, Swansea University, Swansea, SA2 8PP UK; 2 Chelonia Ltd. The Barkhouse, North Cliff, Mousehole, Cornwall, TR19 6PH UK; 3 SEACAMS, College of Science, Swansea University, Swansea, SA2 8PP UK; 4 School of Biological and Marine Sciences, University of Plymouth, Drake Circus, Plymouth, PL4 8AA UK; 5 Department of Life and Environmental Sciences, Bournemouth University, Poole, BH12 5BB UK; 6 Hull International Fisheries Institute, University of Hull, Hull, HU6 7RX UK; 7 Sea Mammal Research Unit, Scottish Oceans Institute, University of St Andrews, Fife, KY16 8LB UK; 8 Vanishing Point Marine, 8 Admiral’s Hard, Stonehouse, Plymouth, PL1 3RJ UK; 9 Marine Scotland Science, 375 Victoria Road, Aberdeen, AB11 9DB UK; 10 Natural England, Sterling House, Dix’s Field, Exeter, EX1 1QA UK; 11 Environment Agency, Riversmeet House, Northway Lane, Tewkesbury, Gloucestershire, GL20 8JG UK; 12 Salar Environmental Services Ltd, Merthyr Mawr Rd, Bridgend, CF31 3NR UK

**Keywords:** Acoustic tags, Passive acoustic monitoring, Vemco, Innovasea, Decoding, C-POD

## Abstract

Aquatic biotelemetry increasingly relies on using acoustic transmitters (‘tags’) that enable passive detection of tagged animals using fixed or mobile receivers. Both tracking methods are resource-limited, restricting the spatial area in which movements of highly mobile animals can be measured using proprietary detection systems. Transmissions from tags are recorded by underwater noise monitoring systems designed for other purposes, such as cetacean monitoring devices, which have been widely deployed in the marine environment; however, no tools currently exist to decode these detections, and thus valuable additional information on animal movements may be missed. Here, we describe simple hybrid methods, with potentially wide application, for obtaining information from otherwise unused data sources. The methods were developed using data from moored, acoustic cetacean detectors (C-PODs) and towed passive receiver arrays, often deployed to monitor the vocalisations of cetaceans, but any similarly formatted data source could be used. The method was applied to decode tag detections that were found to have come from two highly mobile fish species, bass (*Dicentrarchus labrax*) and Twaite shad (*Alosa fallax*), that had been tagged in other studies. Decoding results were validated using test tags; range testing data were used to demonstrate the relative efficiency of these receiver methods in detecting tags. This approach broadens the range of equipment from which acoustic tag detections can be decoded. Novel detections derived from the method could add significant value to past and present tracking studies at little additional cost, by providing new insights into the movement of mobile animals at sea.

## Introduction

Understanding the distribution, movements and migrations of aquatic animals is important for informing issues central to the ecology, conservation and management of their populations, especially when these species are exploited or imperilled (Crossin et al., 2017). Data on animal movements in aquatic ecosystems are also critical for marine planning, for example assessing the effectiveness of marine protected areas (Lea et al., 2016) and for understanding the impact of developments such as renewable energy sites (Reubens et al., 2013; Everley et al., 2016).

**Fig. 1:**
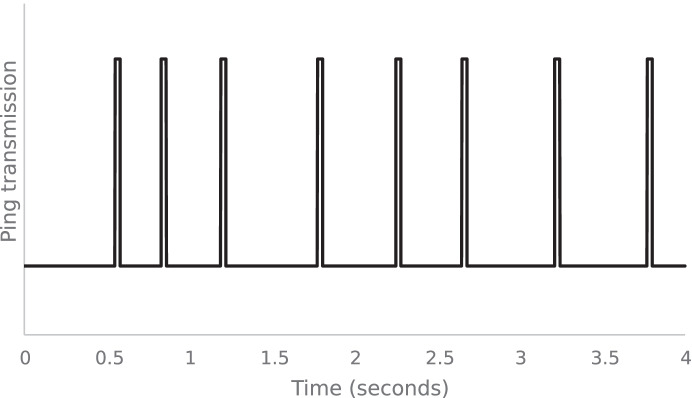
A typical code packet transmission from an acoustic tag. Discrete ‘pings’ are transmitted at a specified frequency with the gap between each ping making up a unique code identifier for the tag

Animal movement data from the marine environment can be derived from various sources, working on their own or in combination (Polagye et al., 2020). These include active acoustic surveys using echosounders for larger organisms such as fish, cephalopods and cetaceans (Williamson et al., 2017), satellite tag tracking (Esteban et al., 2017), catch data (Maunder & Punt, 2004) and mark-recapture studies (Thorrold et al., 2001). All these methods have their limitations and, consequently, marine phase movements of many marine and diadromous species are still poorly understood. In the marine environment, radio transmissions are rapidly attenuated (Lucas & Baras, 2000) but sound propagates well, thus individually coded acoustic transmitters, (‘tags’ hereafter) combined with arrays of fixed passive (receive only) acoustic receivers, are increasingly used to collect data on animal movements (Hussey et al., 2015). These tags emit groups of pings in a discrete packet (Fig. 1), with the timing of pings comprising a code (termed pulse position modulation, PPM) which is decoded by the passive receivers.

As acoustic tags can operate in both freshwater and marine environments, they can be used to track both migratory and non-migratory aquatic animals throughout extensive periods of their lives, especially with technological developments increasing the battery life of some tags to over 3 years (Davies et al., 2020; Drenner et al., 2012; McMichael et al., 2010), which is particularly beneficial for iteroparous diadromous species, enabling the monitoring of movements over multiple spawning cycles.

Acoustic receiver arrays are cost and effort intensive to deploy and maintain. This means that they are generally limited to specific areas and require considerable funding. Many tag detection studies therefore focus on one or, a few, fixed arrays in a limited area, such as an estuary, or ‘fence lines’ or ‘gates’ to try and identify migration direction and pathways, for example, when determining salmon smolt migration paths (Clements et al., 2005). Consequently, wider scale movement information beyond the location of these arrays tends to be limited to occasional recaptures by anglers or commercial fishermen (Lucas & Baras, 2000), or detection in arrays deployed by other researchers (Abecasis et al., 2018). In wider ranging and more mobile species, these large-scale movements may be more important to understanding their ecology than more focused studies. Some more recent studies are also using remote glider vehicles (Zemeckis et al., 2019) and automated unmanned vehicles (AUV) technology to detect tags (Hayes et al., 2013), but these are currently low in number and limited in coverage. Passive acoustic monitoring (PAM) devices are routinely deployed to look at the distributions and densities of cetaceans. In some cases, substantial numbers of devices have been deployed over larger areas; during the SAMBAH project, 300 devices were deployed, with a combined data recording a combined total of over 400 years of acoustic data in the Baltic sea (Carlén et al., 2018). Over 600 C-POD deployments have been carried out around Scotland’s east and west coasts including the mouth of the river Dee in Aberdeenshire over last decade, and this type of data is available from many other areas. Another passive acoustic monitoring device which is widely used in cetacean research is the towed hydrophone array. These are towed behind vessels and usually provide bearing information as well as detections and can also be used to locate vocalising animals through acoustic tracking. The two approaches are complementary; static devices provide excellent temporal coverage while towed arrays surveys can provide spatial coverage. If such underwater noise monitoring devices could be used to detect tagged organisms, then it could potentially provide a powerful tool to understand the movements, dispersal capabilities and the interactions of these species with other groups.

As these tags operate at frequencies within the range of odontocetes echolocation clicks (Mellinger et al., 2007), PAM recorders and detectors used for acoustic detections of odontocetes are generally able to detect pulses produced by acoustic tags. The tags’ output consists of a unique coded audio packet of a known frequency. The time delays between pings within the packet makes up the diagnostic identifier code for that specific tag (Innovasea, 2020b). Thus, for the recordings made on cetacean PAM devices to be most useful, tag transmissions must be both detected and decoded. The aim here was to develop a simple and effective method for decoding detections of acoustic tags recorded on alternative underwater noise monitoring systems, such as C-PODs or F-PODs (generically PODs) and towed hydrophone arrays, including code packet reconstruction to enable the retrieval of tagging data. The objectives were to:

1. Determine codes from acoustic tags can be interpreted using recordings or detections made using third party hardware
2. Develop methods to reprocess archived data so that a range of passive acoustic monitoring data, including historic archived datasets, can be used to detect tag data
3. Assess the accuracy and tag detection range available to underwater noise monitoring systems
4. Investigate the potential to utilise data from a pilot study area

## Material and methods

### Study area and tagged populations

**Fig. 2:**
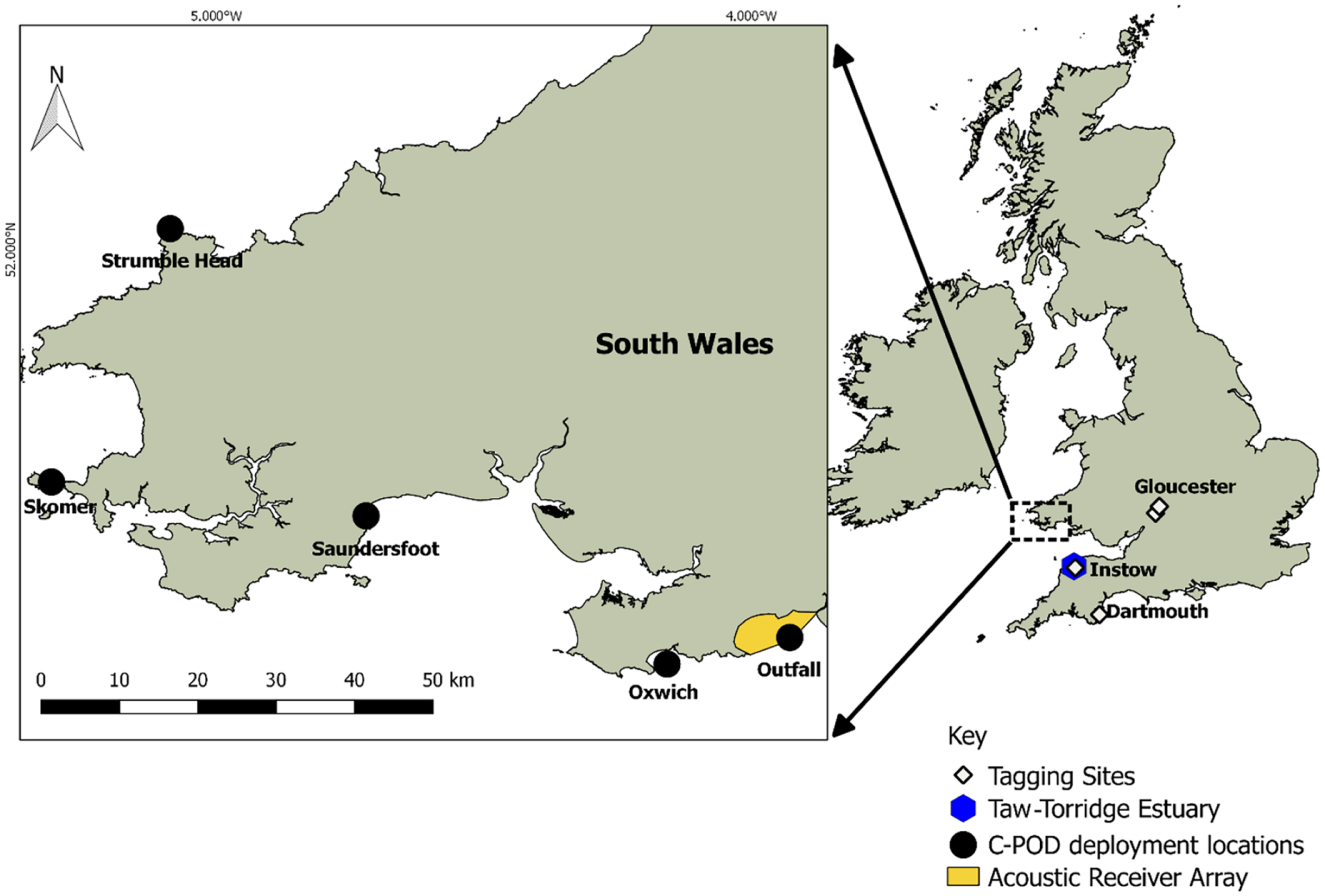
The Bristol Channel study area showing fish tagging locations along with C-POD deployments, towed array surveys in the Taw-Torridge Estuary and the location of the Swansea University Innovasea receiver array, consisting of 29 receivers

In our area of study (Bristol Channel and South West UK, Fig. 2), several studies have tagged fish with 69 kHz Innovasea V9 (formerly Vemco) acoustic tags. Although other attachment methods are possible, including external placement, the size of these tags is such that implantation in the body cavity is typically preferred for fish (Reese et al., 2015; Bolland et al., 2019). During our study period, two projects had animals implanted with Innovasea tags potentially present in the Bristol Channel. The ‘Unlocking the Severn’ team tagged Twaite shad (*Alosa fallax*) and 58 of the 91 tagged fish were known to have left the Severn and entered the Bristol Channel in 2018 and 2019 respectively (Davies et al., 2020). The University of Plymouth University (Stamp et al., 2021) tagged 49 European sea bass (*Dicentrarchus labrax*) in the Taw-Torridge estuary and 51 in the River Dart on the South Coast of England. There is also the possibility that tags from long distance migrants from studies further afield may also be present.

Although this paper focusses on methods for tag detection rather than behaviour, animal welfare, consideration of the possible effect of tagging and external factors such as boat noise are important in interpretation of data of this nature. Both the tagging projects were home office licenced (licence numbers PD6C17B56, P81730EA5) and followed best practice for tag implantation (Bolland et al., 2019, Stamp et al., 2021). Detections in this paper were months or years after initial tagging and acute effects of tagging are unlikely.

### Approach

**Fig. 3:**
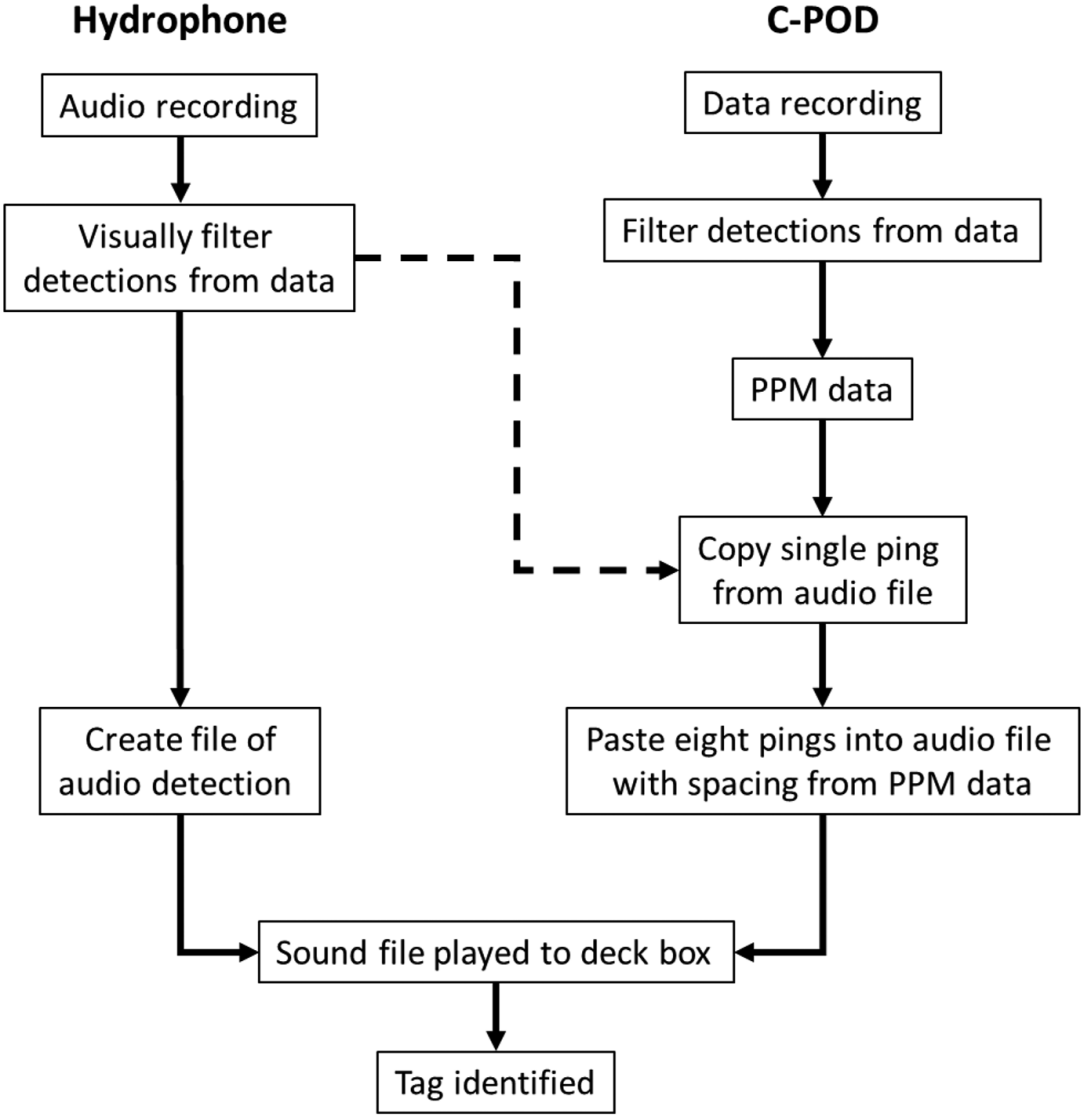
The two routes to decode code packets described. The left-hand side shows the simple playing back of an audio sound file to the decoding deck box. The right-hand side described the reconstruction of a code packet audio file from pulse position modulation (PPM) data using a ping audio snippet copied from a directly recorded audio file

Underwater noise monitoring systems used for detecting cetaceans vary in the extent to which they process data before storage. At one extreme, continuous audio files (.wav) are recorded allowing offline analysis after surveys, others have in built detectors and classifiers and store the time and acoustic characteristics of selected sound types that would include these tags because their characteristics are well within the range of characteristics of transient sounds (clicks) made by cetaceans. A common compromise is to run a general detector of transient ‘clicks’ and store the waveforms of these. The methods presented here focus on two data types, raw audio file and data processed in the receiver to give time-stamped summary data on individual clicks or tonal events. Since the development of the second method requires the first (Fig. 3), the audio recording of tags and their decoding is described first, followed by reconstruction of code packets from processed data. Field data were collected within the Bristol Channel, UK, using three methods:

1. Innovasea acoustic receiver array deployed in Swansea Bay
2. Towed hydrophone array, surveys taken within the Taw/Torridge estuary
3. C-POD deployments from five South and West Wales sites (Swansea Bay, Oxwich Bay, Saundersfoot, Skomer Island and Strumble Head) comprising six individual C-POD deployments over 18 months covering a total of 734 days

### Acoustic tags

Tags available for detection in the Bristol Channel area were Innovasea 69 kHz V9. These tags produce a coded series of 8 pulses at 69 kHz over 3–3.5 s with pulse position modulation (PPM) coding for the lifespan of the tag, typically 1 to 3 years.

### Using raw acoustic signals

Any audio recording with a sampling rate above 140 k samples per second should contain the required information to allow the identification of code packets from tag transmissions. In this study, we used a custom-built 5 channel towed array hydrophone built by Vanishing Point Marine UK for cetacean surveys. Data were recorded through a National Instruments USB Digital Acquisition Unit as.wav files using PAMGuard (V2.01.03) cetacean analysis software (Gillespie et al., 2009). A range testing tag, which transmits a code packet every minute, was suspended from a moored buoy and the array towed in the vicinity of the tag while recording. Towed array surveys were made over 2 days in the Taw/Torridge estuary in North Devon, UK.

Spectrograms from these raw sound files were visually processed to identify and isolate fish tag codes using the Audacity (2.4.2) audio editing suite. The sections of sound files containing these code packets were played in water through Behringer U-PHORIA UMC202HD sound card capable of generating signals at the high frequencies produced by the tags. This drove a high-frequency HS/150 SRD Ltd UK spherical hydrophone placed next to a receiver. The code packets were decoded using an Innovasea VR100-200 decoder connected to the receiver.

### Conversion of C-POD data to acoustic files

To develop a method for the reconstruction of code packets, C-POD passive acoustic monitoring instruments from Chelonia Ltd. were used (Au & Lammers, 2016). These instruments are designed to detect the presence of toothed whales acoustically (Simon et al., 2010). Since the frequency used by acoustic tags, from tens to hundreds of kHz, is within the range used by cetaceans, C-PODs have the capability to detect tag code packets (Mellinger et al., 2007; Nuuttila et al., 2018). The F-POD is a successor to the C-POD and logs over a range of 17–210 kHz compared to 20–160 kHz for the C-POD. The aim here was to therefore develop a simple, quick and effective method for extracting and decoding detections of acoustic tags from data collected for other purposes in formats such as raw audio data or partially processed data such as that stored by C-PODs or F-PODs.

Innovasea tags were identified within the C/FP1 files using F-POD.exe freeware (V1.0.1.16) with an additional function created for this purpose. This ‘Export VEMCO tag data’ function searches for.CP1 or.FP1 files across directories and searches within each file for tag transmissions. That is done by filtering to remove all except 69–72 kHz pings with a minimum duration 5 cycles and searching within this subset for a standard initial inter-ping interval followed by a series of at least 6 more pings. The algorithm is designed to be tolerant of some multipath propagation of pings, and produces, for each packet of pings, a series of ping times and a score that is high if the data is clean and falls if there are many multipath or ‘stray’ pings or if the ping amplitudes vary greatly. Because these data files already contain frequencies of ‘clicks’, the process is fast, taking generally less than 20 s per year of acoustic recording. The results are in a simple text format that can be pasted into a spreadsheet.

An audio recording from the towed array, which included a tag code packet, was opened in the audacity audio editing package (V 2.1.0) and a baseline file of a 5-s duration was created using background noise from this file at the maximum project rate available of 384,000 Hz.

**Fig. 4:**
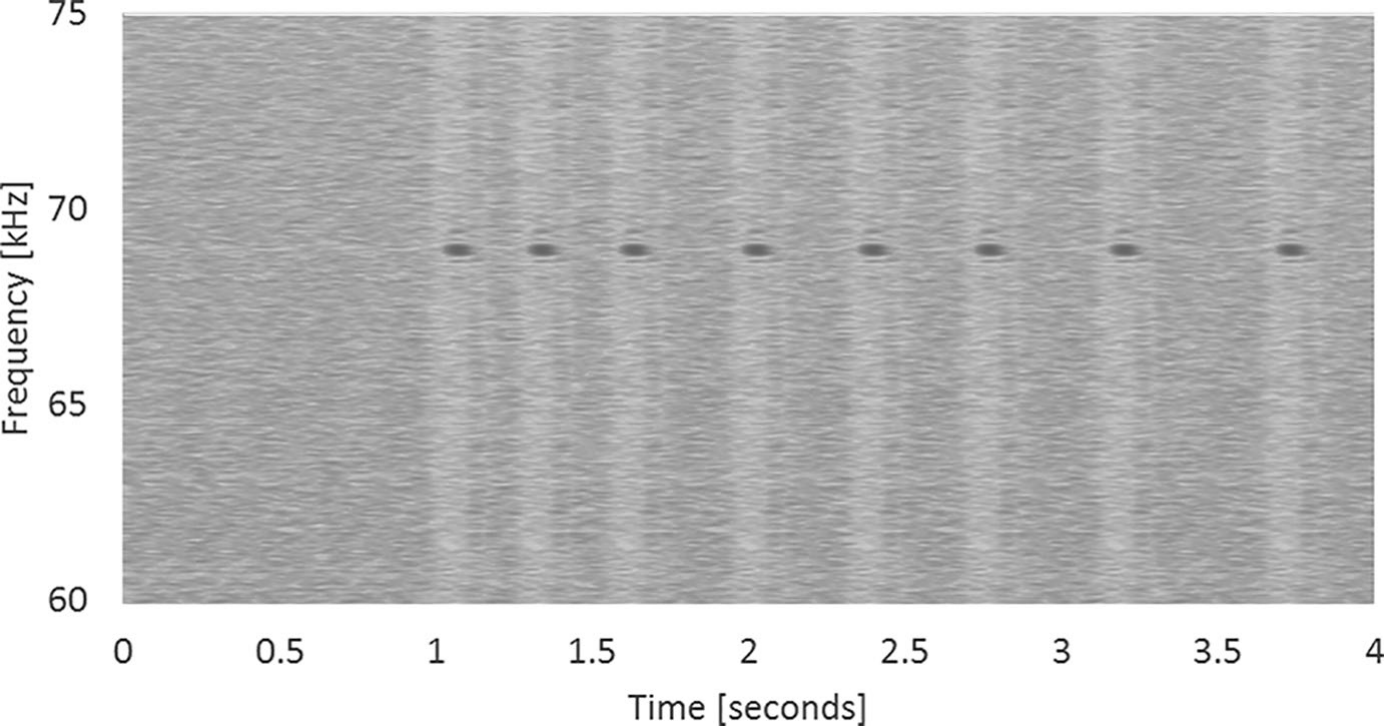
A spectrogram of a reconstructed code packet from a C-POD detection derived from F-POD.exe outputs

A single ping from the identified packet was then copied from the audio file and into a ‘tag reconstruction’ file at *T* = 1000 ms. This delay was included to reduce the likelihood of acoustic artefacts arising from the start of the file during playback. Each of the following seven pings were then copied into the same file using the ping intervals derived from the C-POD data to reconstruct the code packet from the tag transmission. The resulting audio (Fig. 4) was then output as a.wav file to be played to the Innovasea deck box through the same method as the raw recorded data. Construction of a code packet file took less than 1 min, and further automation to allow batch processing is in development.

### Accuracy test

A series of 16 Innovasea V7 tags were lowered in turn into Swansea marina within 5 m of a C-POD and next to the receiver of an Innovasea deck box with each tag being removed before the next was lowered. The V100-200 deck box recorded the time at which each tag transmitted a code packet and also confirmed and cross-checked the identity of the tag. Data were offloaded from the C-POD and reconstructed acoustic files created as above, before being played back to the deck box via the hydrophone and receiver.

### Range testing

In order to understand the potential value of alternative methods of tag detection, the range of detection using Chelonia Ltd. C-PODs was compared to Innovasea acoustic VR2W and VRTx receivers. There are a number of different environmental factors which can affect the detection range of acoustic receivers (Ammann, 2020; Heupel et al., 2008), and so we undertook paired range tests to allow the relative performance of the two types of equipment to be compared.

Range testing was conducted in the coastal waters of Swansea Bay, south Wales. Four manufacturer-calibrated C-PODS (cetacean-porpoise detectors) were deployed on a GPS marked buoyed mooring at 3 m, 6 m, 9 m and 12 m from the seabed, in approximately 20 m of water. On a separate GPS marked mooring, four Innovasea acoustic receivers were deployed at the same depth intervals. The hydrophones of both the C-POD and Innovasea receivers were aligned at each depth. The deployments had two trawl floats attached to the mooring line to ensure buoyancy. To compare the detection range, an Innovasea V9 coded range testing tag, which transmits a code package once every ten seconds was attached to a third mooring line, at 6 m from the seabed. Deployments of the range testing tag, using approximately 50 kg of weight, were made at exact GPS coordinates, the rapid sinking of which minimised displacement of the moorings from the intended location, with an assumed variation of no more than 10 m.

The test tag was deployed at an equal distance between Innovasea and C-POD receivers at ranges of 100 m, 200 m, 300 m, 350 m, 400 m, 450 m and 500 m. At each distance, the vessel deployed the tag, moved away from the mooring and remained in neutral to ensure the underwater noise from the vessel did not interfere with the ability of the equipment to detect the tag. After 10 min, the vessel returned to the test tag mooring and towed it to the next distance interval. This was repeated over 2 days using the same GPS coordinates each time, to give a total of three replicates.

*F-POD.exe* software V1.0.0.05 was used to identify code packet detections. At each distance, the data recorded on the C-POD for the 10-min time period was also manually checked for tag detections to determine the range of each C-POD. Data from each Innovasea receiver were downloaded using *VUE* software V2.6.2(0046). Data were inspected for test tag detections at each of the measured distances for comparison with the C-PODs.

## Results

### Direct recording

**Fig. 5:**
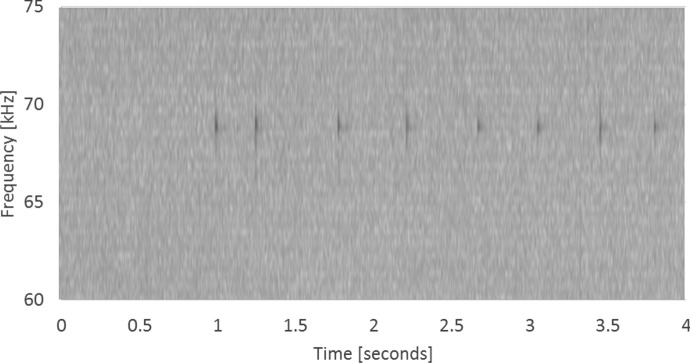
A spectrogram of a coded acoustic packet from an Innovasea acoustic fish tag showing a tag code packet of approximately 3-s duration

Playback of recorded audio files from the towed acoustic array in the vicinity of a deployed Innovasea range testing tag showed clear detection of the code packet (Fig. 5) and correctly identified the specific tag that the recording was made from. Towed array trials at three locations showed considerable range variation between sites with detection ranges of 400 m in Swansea Bay, 200 m in the Taw/Torridge estuary and around 50 m downstream of the Severn Bridge.

Data from a towed acoustic survey of the Taw-Torridge estuary also provided code packets from three tagged wild fish in the environment. Tags identified through playback to the Innovasea deck box coincided with tags implanted into bass (*Dicentrarchus labrax*) with concurrent detections of these fish on Innovasea receivers deployed in the estuary by the I-BASS project.

### C-POD reconstruction–accuracy

C-POD recordings of Innovasea V9 tags deployed in Swansea Marina were correctly identified after their reconstructed code packets were played to the deck box via hydrophone. No incorrect tag identifications were made.

## C-POD reconstruction–wild recordings

A total of 26 code packets identified as tags were extracted from C-POD data. All sites except the Skomer deployment contained acoustic tag detections identifying six individual fish (Table 1), with tag codes corresponding to fish tagged in the Bristol Channel, or in one instance the Dart estuary on the southern side of the South West England peninsular. Of these, four were Twaite shad (*Alosa fallax*) that had been tagged in the upper Severn (Davies et al., 2020) and two bass (*Dicentrarchus labrax*) tagged in North Devon by the University of Plymouth under the i-BASS project (I-BASS, n.d.). The bass (1602–25,217) tagged on the Dart on 10/08/2018 was detected at both Saundersfoot in June 2019 and at Strumble Head in October and November 2019, while the bass 1602–25,217 detected using the towed hydrophone array in North Devon was also detected on the other side of the Bristol Channel in Swansea Bay using Swansea University’s Innovasea receiver array. The shad 1602–26,317 was detected on C-PODs and additionally the receiver array (Table 1).

**Table 1:** Detections of tagged fish in C-POD and towed hydrophone array surveys in the Bristol Channel in 2018–2019 ordered by date

**Method**	**Location**	**Date**	**Tag ID**	**Species**	**Tagged**
**C-POD**	Swansea Bay Outfall	16/06/2018**	1602–26,278	*Alosa fallax*	Gloucester
**C-POD**	Oxwich Bay	01/07/2018	1602–25,163	*Dicentrarchus labrax*	Taw/Torridge
**C-POD**	Oxwich Bay	05/07/2018	1602–25,163	*Dicentrarchus labrax*	Taw/Torridge
**C-POD**	Oxwich Bay	18/07/2018**	1602–25,163	*Dicentrarchus labrax*	Taw/Torridge
**C-POD**	Swansea Bay Outfall	22/07/2018	1602–26,319	*Alosa fallax*	Gloucester
**C-POD**	Swansea Bay Outfall	10/09/2018	1601–47,652	*Alosa fallax*	Gloucester
**C-POD**	Oxwich Bay	12/08/2018**	1602–25,163	*Dicentrarchus labrax*	Taw/Torridge
**C-POD**	Swansea Bay Outfall	10/09/2018	1602–26,317*	*Alosa fallax*	Gloucester
**C-POD**	Oxwich Bay	03/10/2018	1602–25,163	*Dicentrarchus labrax*	Taw/Torridge
**C-POD**	Saundersfoot	16/06/2019**	1602–25,217	*Dicentrarchus labrax*	Dart
**Towed array**	Taw-Torridge	21/06/2019	1602–25,151*	*Dicentrarchus labrax*	Taw/Torridge
**Towed array**	Taw-Torridge	09/07/2019	1602–25,151*	*Dicentrarchus labrax*	Taw/Torridge
**Towed array**	Taw-Torridge	09/07/2019	1602–25,169	*Dicentrarchus labrax*	Taw/Torridge
**Towed array**	Taw-Torridge	09/07/2019	1602–25,176	*Dicentrarchus labrax*	Taw/Torridge
**Towed array**	Taw-Torridge	09/07/2019	1602–25,176	*Dicentrarchus labrax*	Taw/Torridge
**Towed array**	Taw-Torridge	10/07/2019	1602–25,169	*Dicentrarchus labrax*	Taw/Torridge
**C-POD**	Strumble Head	14/10/2019	1602–25,217	*Dicentrarchus labrax*	Dart
**C-POD**	Strumble Head	03/11/2019	1602–25,217	*Dicentrarchus labrax*	Dart
**C-POD**	Strumble Head	04/11/2019	1602–25,217	*Dicentrarchus labrax*	Dart

^*^Also detected in Innovasea receiver array in Swansea Bay; ^**^Multiple detections during the day

## Range test–C-POD vs. receivers

Table 2 shows the relative range at which Innovasea acoustic receivers and C-PODs were able to detect an Innovasea acoustic test tag in field deployments. No additional detections were identified from manual checking of the C-POD data compared to those detected automatically. In all cases, the range of the Innovasea receiver was greater than that of the C-POD, with the former consistently detecting tags at a range of 450 m and the latter having a range of between 100 and 200 m with much reduced frequency of detection within the ten minutes of sampling. C-PODs and Innovasea acoustic receivers both showed increased detection range when deployed further from the seabed than those near to it.

**Table 2:** Detection range of an Innovasea test tag using (top) Innovasea acoustic receivers and (bottom) C-PODS. x denotes that the instrument at that depth had detected the tag one or more times during the ten minutes that the tag was deployed. Measurements are distance from seabed rather than depth in water

**Innovasea**												
Distance	3 m			6 m			9 m			12 m		
	Run #			Run #			Run #			Run #		
	1	2	3	1	2	3	1	2	3	1	2	3
100 m	x	x	x	x	x	x	x	x	x	x	x	x
200 m	x	x	x	x	x		x	x		x	x	
300 m	x	x		x	x		x	x		x	x	
350 m	x	x	x	x	x	x	x	x	x	x	x	
400 m	x	x			x	x	x	x	x		x	
450 m		x			x	x	x	x	x		x	
500 m							x		x			
**C-POD**												
Distance	3 m			6 m			9 m			12 m		
	Run #			Run #			Run #			Run #		
	1	2	3	1	2	3	1	2	3	1	2	3
100 m		x	x	x		x	x	x	x	x	x	x
200 m	x						x	x		x		
300 m												
350 m												
400 m												
450 m												
500 m												

## Discussion

An increasing number of instruments are being deployed in marine and freshwater environments that are capable of processing and recording high frequency sounds (Howe et al., 2019; Kessel et al., 2014; Zemeckis et al., 2019). These represent a large reservoir of untapped data which could be analysed for acoustic tag signatures. Here, we show how to make use of these data to gain added value in the detection of acoustic tags implanted into aquatic animals. We have demonstrated a low cost, simple method to support acoustic tag surveys by identifying tags both from direct acoustic recordings and by reconstructing code packets from processed C-POD data. Conceptually, any passive acoustic device that records sounds or ping events at the frequencies used by a target tag type could be used to allow reconstruction of tag code packets and decoding through this method.

Depending on the design of tags used, different characteristics may be used to identify code packets in a dataset and could include ratios of amplitudes and frequencies between concurrent pings and code delay checksums. These considerations are important for the filtering of detections of code packets within datasets but are not applicable to the decoding of that packet through the deck box. Given a simple level of automation, even if robust filtering were not possible, batch processing of large numbers of potential tags should be possible, which would reject non-tag detections and only identify those tags which fulfil the manufacturer’s quality criteria for detections.

Arrays of acoustic receivers are designed to understand fine-scale movements to investigate subjects such as localised habitat usage and site fidelity (Dahl & Patterson, 2020; Huber & Carlson, 2020), but more dispersed detections could provide key information on issues such as species ranges, foraging and migration. This is particularly important for managing species including migratory and marine fish, as can be seen by the bass tagged on the south English coast detected in Welsh waters this study. Even large, well-funded, studies may still have considerable data gaps. Arrays are almost always geographically clustered and so large expanses are present where no data on the movement of tagged animals is available (Hussey et al., 2015; McAuley et al., 2017). Tagged animals moving outside of their expected ranged would also not be detected. In some cases, these issues can be ameliorated by sharing data and tag codes with groups running similar arrays (Davies et al., 2020), but this still requires deployments of acoustic receivers capable of decoding the tags.

Our method requires the use of a proprietary deck box, although hardware manufacturers could, if they chose, offer a software-based solution which would allow a more robust and reliable approach. This is particularly true as the code spaces used by tags, which have a known shelf life are routinely updated by the manufacturer (Innovasea, 2020a). Filtering parameters used in the current dataset therefore may not be universal even for this system, and will certainly not be for systems from other manufacturers. Although these diagnostic parameters can be determined empirically by analysing audio recordings, having direct confirmation of these from the manufacturer would be more efficient. For example, analysis of the data for Innovasea tags showed a consistent 0.280-s gap between the first and second pings with a frequency based around 69 ± 2 kHz, and a total code packet length of 3–3.5 s for the eight pings in the packet. Filters in the F-POD software were setup based on this and consistently produced tag detections which decoded to tags known to be implanted in local fish; however, confirmation of these timings and hidden diagnostic patterns could easily be provided by manufacturers and would increase both accuracy and the range of tags which could be filtered for automatically. There would also be several advantages to the establishment of a central repository of deployed tag codes, which could be maintained by the manufacturer, following a similar principle to that of the European tracking network (NTN) (Abecasis et al., 2018).

In field-based testing, the detection range and detection rate of proprietary acoustic receivers was, as expected, considerably higher than that of the C-POD systems which are designed for much broader spectrum acoustic monitoring. Conditions such as sediment transport, water depth, topography, wind speed, wave height, precipitation and turbidity have considerable impact on variation in detection range (Ammann, 2020; Heupel et al., 2008; Sostres Alonso & Nuuttila, 2015), as can be seen in the large difference between the ranges seen in the first two deployments and the third, which was taken on the following day. The difference in range observed between receivers and C-PODs would be expected as the hardware and software of proprietary receivers are specifically designed to be sensitive at the frequency at which the tag is transmitting (Vemco, 2016), whereas C-PODs are designed to detect pulsed sounds over much larger frequency band. Nevertheless, as evidenced by our own ‘wild’ detections, the effective range of 100–200 m seen using C-PODS represents a large enough area to pick up fish detections regularly, particularly if placed near features which may aggregate fish. Also, these reduced ranges can be accounted for in experimental designs (Brownscombe et al., 2020).

During this preliminary trial, we made detections in the towed array recordings by scanning spectrograms by eye. There is good scope for making efficient automated detectors using some of the tools already within PAMGuard which could analyse raw audio data from any device recording in a compatible format (Pamguard, 2017). In addition, multi-element towed arrays used in conjunction with software such as PAMGuard provide information on the location and bearing of detections (Gillespie et al., 2009) which could be used to manoeuvre the boat closer to an animal after a weak detection so that good signal to noise recordings could be made for decoding.

For POD data, the automated search processed a year’s worth of data in under twenty seconds, and found all the total detections found by the automated plus manual search. Cumulatively, many hundreds of years of POD data exist from cetacean monitoring projects, with the Baltic Sea having amassed a combined total of 400 years of data from the SAMBAH project alone (Carlén et al., 2018; SAMBAH, 2016). This is a substantial time saving in data processing, and an automated code packet reconstruction process is being developed. This, in conjunction with the automated POD processing and time-stamped logging of code detections from a deck box would allow fully automated end-to-end processing of POD data.

## Conclusions

In our surveys, we reprocessed data from C-PODs and towed acoustic hydrophone arrays and detected tagged fish on most deployments. Despite the limited extent, both spatially and temporally, of these deployments, 9 individual fish were detected, some multiple times at one C-POD or across more than one C-POD. In some cases, fish detected were tracked by other methods; in others these are the only ‘at sea’ detections of the individuals. This reveals that detections from this type of data can be provided that would not otherwise be available and may often be in areas remote from targeted receiver deployments, adding high value these studies. This preliminary study supports the benefit of an examination of PAM data from a wider spatial and temporal scale to explore the effectiveness of a wider application of these methods. Clearly, third party passive acoustic detections cannot replace bespoke receiver arrays, but they can complement these arrays to provide data from areas and times where receivers are not deployed. This added value can be obtained for little time and effort from existing datasets, and the fact that these deployments are often not in the ‘target’ area may enhance their value and contribution to fisheries management.

## Data Availability

The datasets generated during and/or analysed during the current study are available from the corresponding author on reasonable request.

## Code availability

Custom code created for the current study is available from the corresponding author on reasonable request.
